# Gene duplication of type-B ARR transcription factors systematically extends transcriptional regulatory structures in *Arabidopsis*

**DOI:** 10.1038/srep07197

**Published:** 2014-11-26

**Authors:** Seung Hee Choi, Do Young Hyeon, ll Hwan Lee, Su Jin Park, Seungmin Han, In Chul Lee, Daehee Hwang, Hong Gil Nam

**Affiliations:** 1 Department of Life Sciences, POSTECH, Pohang, Gyeongbuk 790–784, Republic of Korea; 2 School of Interdisciplinary Bioscience and Bioengineering, POSTECH, Pohang, Gyeongbuk 790–784, Republic of Korea; 3 Center for Plant Aging Research, Institute for Basic Science (IBS), Daegu 711–873, Republic of Korea; 4 Department of New Biology, Daegu Gyeongbuk Institute of Science & Technology (DGIST), Daegu 711–873, Republic of Korea

## Abstract

Many of duplicated genes are enriched in signaling pathways. Recently, gene duplication of kinases has been shown to provide genetic buffering and functional diversification in cellular signaling. Transcription factors (TFs) are also often duplicated. However, how duplication of TFs affects their regulatory structures and functions of target genes has not been explored at the systems level. Here, we examined regulatory and functional roles of duplication of three major ARR TFs (ARR1, 10, and 12) in *Arabidopsis* cytokinin signaling using wild-type and single, double, and triple deletion mutants of the TFs. Comparative analysis of gene expression profiles obtained from *Arabidopsis* roots in wild-type and these mutants showed that duplication of ARR TFs systematically extended their transcriptional regulatory structures, leading to enhanced robustness and diversification in functions of target genes, as well as in regulation of cellular networks of target genes. Therefore, our results suggest that duplication of TFs contributes to robustness and diversification in functions of target genes by extending transcriptional regulatory structures.

Many of redundant genes are stably maintained in the genome^1^,^2^,^3^. The 'redundant genes' refers to homologous genes with functional overlaps. The persistence of redundant genes has been a key question in the evolution of the genome. Redundant genes are enriched significantly in signal transduction pathways, as well as developmental and metabolic pathways^4^. Genetic redundancy is canonically known to provide genetic buffering and functional divergence^1^,^5^,^6^. Recently, the stable preservation of genetically redundant copies was suggested to provide selective advantages in cellular signaling system by forming negative feedback loops through their reciprocal regulation and thereby achieving robustness and evolvability of cellular signaling systems^4^,^7^.

In cellular signaling pathways, in addition to kinases^8^,^9^,^10^, transcription factors (TFs) are often duplicated, thereby affecting their downstream transcriptional regulatory networks. For example, in *C. albicans*, the genes encoding LYS TFs (LYS14, 142, 143, and 144) are duplicated^11^. Due to their sequence homology, they share common target genes, which can enhance robustness in regulation of their functions (genetic buffering). On the other hand, through gene duplication, they acquired different DNA binding motifs and/or also different associations with cofactors, thereby controlling different sets of target genes (functional diversification). However, how duplication of TFs with functional overlaps systematically establishes common and different downstream target genes in their transcriptional regulatory networks, thus leading to robustness and diversification in functions of target genes, respectively, has not been explored at the systems level.

Cytokinin is a plant hormone that regulates a broad spectrum of plant physiologies, such as cell division, growth, and senescence, by controlling expression of thousands of downstream genes^12^,^13^,^14^,^15^. In the cytokinin signaling pathway, type-B response regulators (ARRs) act as primary TFs that regulate thousands of target genes involved in the responses to cytokinin^16^,^17^. *Arabidopsis* has 11 type-B ARRs with high sequence similarities in receiver and/or transcription activation domains^18^. Of the 11 TFs, ARR1, 10, and 12 are considered to be essential in that the triple mutant of type-B *ARR1, 10, 12* largely abolishes the cytokinin-dependent gene expression and physiological effects^19^,^20^,^21^. Here, we thus analyzed how duplication of the three major type-B ARRs affects their transcriptional regulator structure and functions of their target genes at systems level. For this analysis, we used wild-type (WT) and single, double, and triple deletion mutants of *ARR1, 10*, and *12*, as well as gene expression profiling of WT and these mutants.

## Results and Discussion

### Duplication of TFs systematically extends regulatory structures for target genes

Duplication of TFs can confer an extension of transcriptional regulatory structures for target genes by providing new regulatory relationships between duplicated TFs and new or old target genes. Duplicated TFs can have shared or different target genes, and target genes can also require multiple or only one of duplicated TFs. To examine the nature of the extension in the regulatory structure, we performed gene expression profiling of *Arabidopsis* root tissues obtained from wild-type (WT) and deletion mutants of three type-B *ARR1*, *10*, and *12*. To distinguish whether target genes require one or multiple of ARR1, 10, and 12, we further generated gene expression profiles from single (*arr1*, *10*, and *12*), double (*arr1/10*, *arr1/12*, and *arr10/12*), and triple (*arr1/10/12*) deletion mutants of *ARR1*, *10*, and *12*.

**Figure 1:**
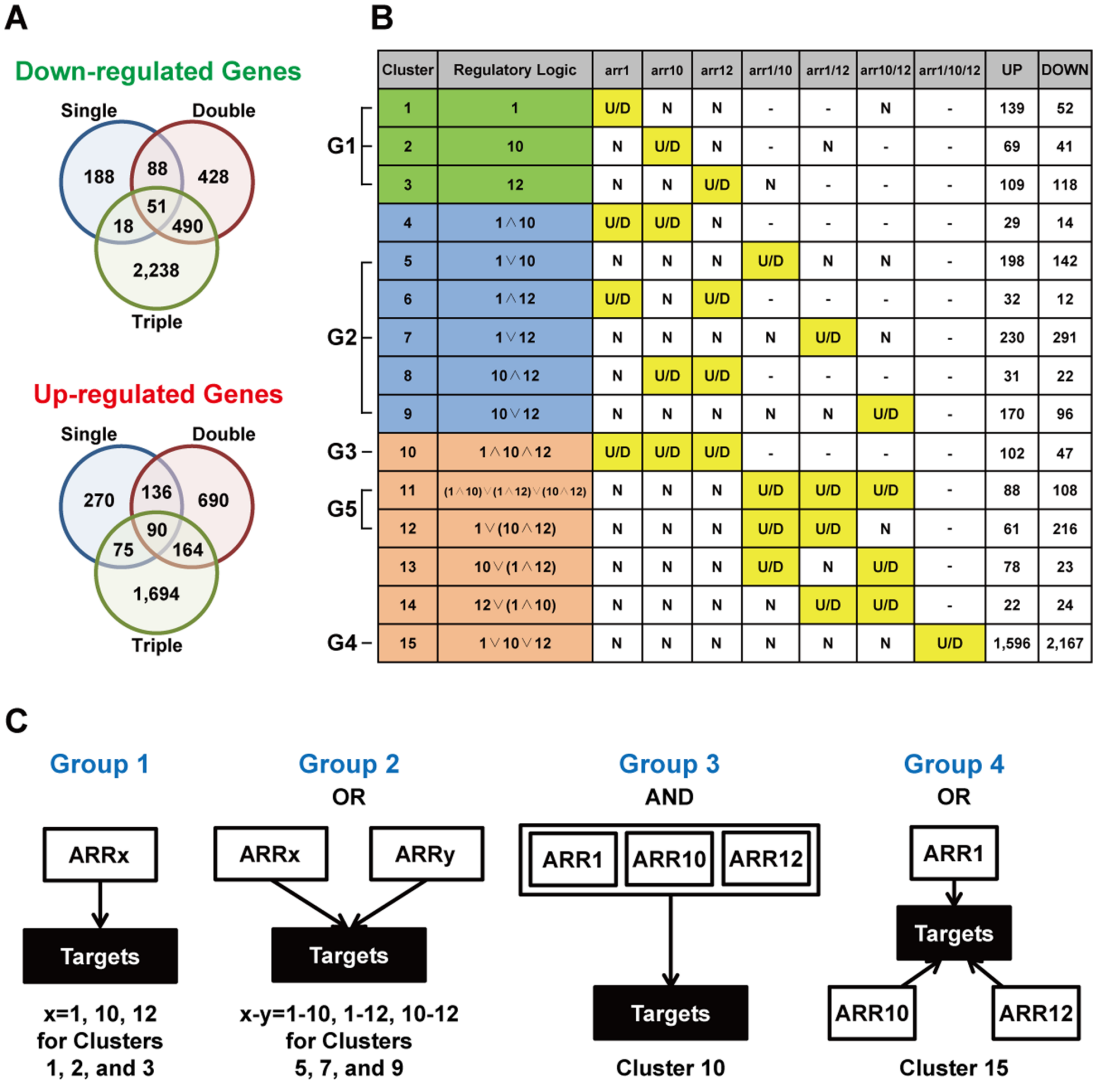
Duplication of *ARR1*, *10*, and *12* extends regulatory structures. (A) Venn diagrams of the genes showing down- (upper) and up- (lower) regulation in single, double, and triple mutants of *ARR1*, *10*, and *12*, compared to WT. (B) Regulatory structures inferred from differential expression patterns in the mutants. For each cluster of the DEGs, the regulatory structure was presented as the regulatory logic. Symbols of ‘∧' and ‘∨' represent ‘AND' and ‘OR' logics, respectively. The numbers of the up- and down-regulated genes for each regulatory logic are also presented. U/D, up- or down-regulation; N, no change. (C) Four major groups of the regulatory structures. Group 1, Clusters 1–3; Group 2, Clusters 5, 7, and 9; Group 3, Cluster 10; and Group 4, Cluster 15.

We first examined target genes regulated by duplicated TFs by identifying differentially expressed genes (DEGs) in the following seven comparisons using an integrative statistical method previously reported^22^: 1–3) *arr1* versus WT, *arr10* versus WT, and *arr12* versus WT; 4–6) *arr1/10* versus WT, *arr1/12* versus WT, and *arr10/12* versus WT; and 7) *arr1/10/12* versus WT. From these comparisons, 916 DEGs (571 up-regulated and 345 down-regulated genes) were identified in single mutants (Comparisons 1–3), 2,137 DEGs (1,080 up-regulated and 1,057 down-regulated genes) in double mutants (Comparisons 4–6), and 4,820 DEGs (2,023 up-regulated and 2,797 down-regulated genes) in the triple mutant (Comparison 7) (Fig. 1A). The increase in the numbers of DEGs from single, double to triple deletion mutants indicates that duplication of the ARR TFs increased the size of target genes.

To examine the regulatory structure between duplicated TFs and the target genes (i.e., the DEGs), we first categorized the DEGs into Clusters 1–15 (Fig. 1B; Table S1) based on their differential expression (up- or down-regulation) in the mutants of *ARR1*, *10*, and *12* (Methods). For each cluster, we then mapped an AND or OR regulatory structure that was inferred from differential expression of the DEGs in single, double, and triple mutants. For example, 291 genes in Cluster 7 were down-regulated in *arr1/12* double mutants, but not changed in expression in any of single mutants (*arr1*, *10*, and *12*), nor in *arr1/10* and *arr10/12*. These data indicate that the 291 genes were regulated by either ARR1 or ARR12 (1∨12 in Fig. 1B). Also, 102 genes in Cluster 10 were up-regulated in all three single mutants (*arr1*, *10*, and *12*), suggesting that all the three ARRs were required to suppress the expression of these genes (1∧10∧12 in Fig. 1B).

From this analysis, we found total 15 distinct regulatory structures (Clusters 1–15 in Fig. 1B; Fig. S1) for up- or down-regulated genes. Cluster 1, 2, or 3 represents the regulatory structure defined by a single TF. However, Clusters 4–15 were defined by combinations of duplicated TFs and represent the regulatory structures additionally acquired through duplication of TFs. Of the 15 clusters, we selected the 10 clusters with top 50^th^ percentile of up- or down-regulated cluster sizes (Methods). We then grouped them into five groups based on the similarity in their regulatory structures (Fig. 1B; Fig. S1): **Group 1**) Clusters 1–3 regulated uniquely by individual TFs; **Group 2**) Clusters 5, 7, and 9 with OR logics of two TFs; **Group 3**) Cluster 10 with AND logic of three TFs; **Group 4**) Cluster 15 with OR logic of three TFs; and **Group 5**) Clusters 11–12 with mixed AND and OR logics of three TFs. Of these five groups, we focused on Groups 1–4 in the following analyses (Fig. 1C) because Group 5 included Cluster 11 [(1∧10)∨(1∧12)∨(10∧12)] that embedded the AND logics of two TFs (e.g., 1∧10) not significant in their cluster sizes and Cluster 12 [1∨(10∧12)] whose parallel structures with the similar logics (Clusters 13–14) were not significant. These data indicated that duplication of TFs extensively extended the regulatory structure, as well as the size of target genes as shown in Fig. 1A.

### Extended regulatory structures contribute to robustness and diversification in transcriptional regulation of target genes

**Figure 2:**
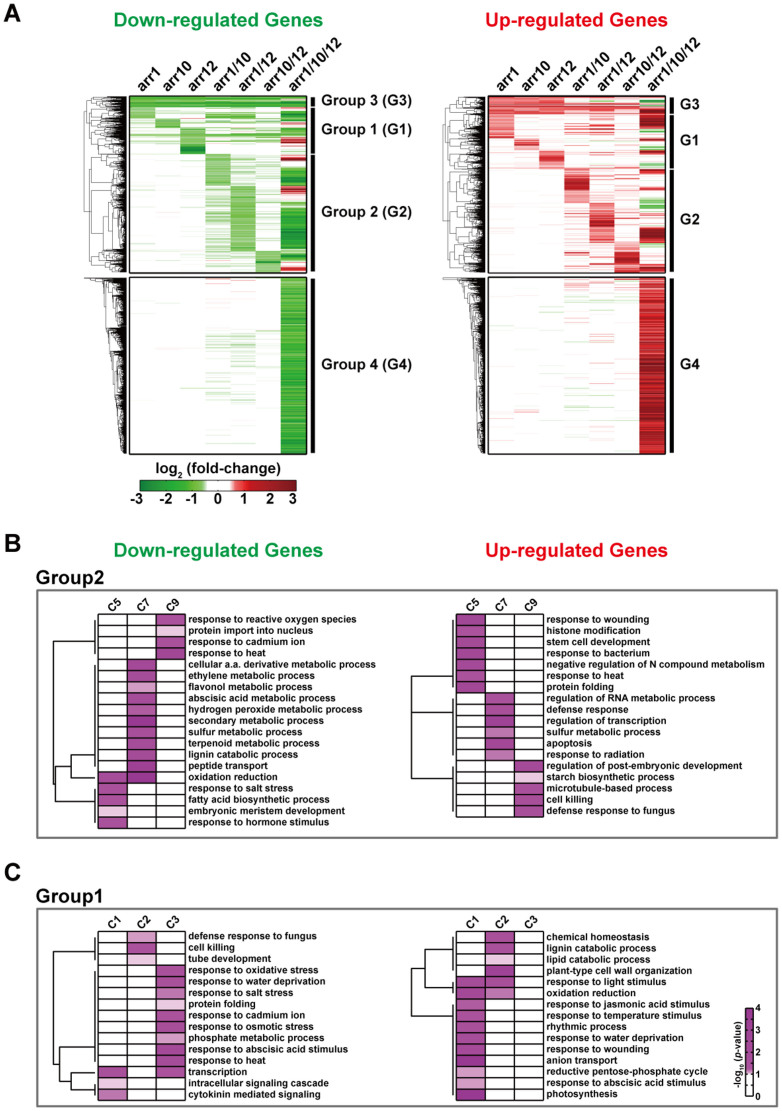
Extended regulatory structures contribute to robustness and diversification in functions of target genes. (A) Down- (left) and up-regulated genes (right) in the four major groups of the DEGs (Groups 1–4) in the single, double, and triple mutants of *ARR1*, *10*, and *12*, compared to WT. Green and red colors represent down- and up-regulation, respectively. Color bar shows the gradient of log_2_-fold-changes of gene expression levels between the mutant and WT. (B–C) GOBPs represented by the down- (left) and up-regulated genes (right) in Groups 2 (B) and 1 (C). The color bar represents the gradient of -log_10_ (*p*-value) where *p*-value is the significance of the GOBPs being enriched by the genes in each group, which was computed from DAVID software. The genes (A) and GOBPs (B–C) in the heat maps were clustered using the log_2_-fold-changes and -log_10_ (*p*-value), respectively, by a hierarchical clustering method (average linkage and Euclidean distance as similarity measure). Clusters 1–3, C1–3; Clusters 5, 7, and 9, C5, C7, and C9; and Groups 1–4, G1–G4.

Gene duplication was previously reported to provide robustness and also diversification in functions of duplicated genes^1^,^5^,^6^. This previous finding led to a notion that duplication of the ARR TFs might provide robustness and/or diversification in transcriptional regulation of downstream target genes. To test this notion, we examined Groups 1–4 representing the major transcriptional regulatory relationships in the extended structure for up- and down-regulated genes (Fig. 2A). Group 2 or 4 with OR logics included the genes redundantly regulated by two or three ARR TFs, respectively, indicating that duplication of the ARR TFs conferred robustness in transcriptional regulation of these genes (Fig. 1B). On the other hand, Clusters 1–3 in Group 1 included the genes uniquely regulated by the individual ARR TFs, respectively, indicating that duplication of the ARR TFs resulted in diversification in target genes of ARR TFs and thereby transcriptional regulation of the target genes (Fig. 1B). Moreover, the genes redundantly regulated by the two ARR TFs in Group 2 were further diversified into Clusters 5, 7, and 9, indicating that Group 2 provides both robustness (OR logics) and further diversification (multiple different OR logics). All these data suggest that duplication of the ARR TFs contributes to robustness and diversification in transcriptional regulation of target genes.

### Extended regulatory structures contribute to robustness and diversification in cellular functions of target genes

The extended regulatory structures by duplication of TFs provided robustness and diversification in transcriptional regulation of the downstream target genes. This further led to a notion that duplication of TFs can then contribute to robustness and diversification in cellular functions of their downstream target genes through the extended regulatory structures. To test this notion, we performed the enrichment analysis of gene ontology biological processes (GOBPs) for the genes in the clusters of Groups 1–4 using DAVID software^23^ (Fig. S2; Table S2 and S3). The GOBPs represented by the down- and up-regulated genes in Group 2 (Clusters 5, 7, and 9) included the processes mainly related to stress responses (responses to heat, reactive oxygen species, cadmium ion, etc.), metabolism (ethylene, abscisic acid, terpenoid, and fatty acid metabolic processes), and defense responses (apoptosis, response to bacterium, and defense response) (Fig. 2B). These GOBPs corresponded to cellular functions redundantly regulated by two of the three ARR TFs. Similarly, the GOBPs represented by Group 4 are cellular processes redundantly regulated by the three ARR TFs (Cluster 15 in Fig. S2). These data indicate that duplication of the ARR TFs contributes to robustness in cellular functions of the target genes.

To examine whether duplication of the TFs contributes to diversification in cellular functions of the target genes, we then compared the GOBPs represented by Clusters 1–3 uniquely regulated by the individual ARR TFs. The GOBPs represented by Clusters 1–3 largely differed from each other, indicating that duplication of the ARR TFs contributed to diversification in cellular functions of their target genes (Fig. 2C). For example, for the down-regulated genes, Cluster 1 were associated with cytokinin mediated signaling, Cluster 2 with defense responses (defense response to fungus and cell killing), and Cluster 3 with stress responses (responses to oxidative stress, salt stress, water deprivation, etc.). Moreover, we showed above that the genes redundantly regulated by the two ARR TFs in Group 2 were further diversified into Clusters 5, 7, and 9. Comparison of the GOBPs represented by Clusters 5, 7, and 9 revealed that a number of GOBPs were uniquely represented by the individual clusters, respectively (Fig. 2B). For example, for down-regulated genes, response to hormone stimulus, secondary metabolic process, and response to reactive oxygen species were uniquely represented by Clusters 5, 7, and 9, respectively. All these data suggest that the extended regulatory structure by duplication of the ARR TFs contributes to robustness and diversification in cellular functions of target genes.

### Extended regulatory structures contribute to robustness and diversification in regulation of hormone signaling networks

**Figure 3:**
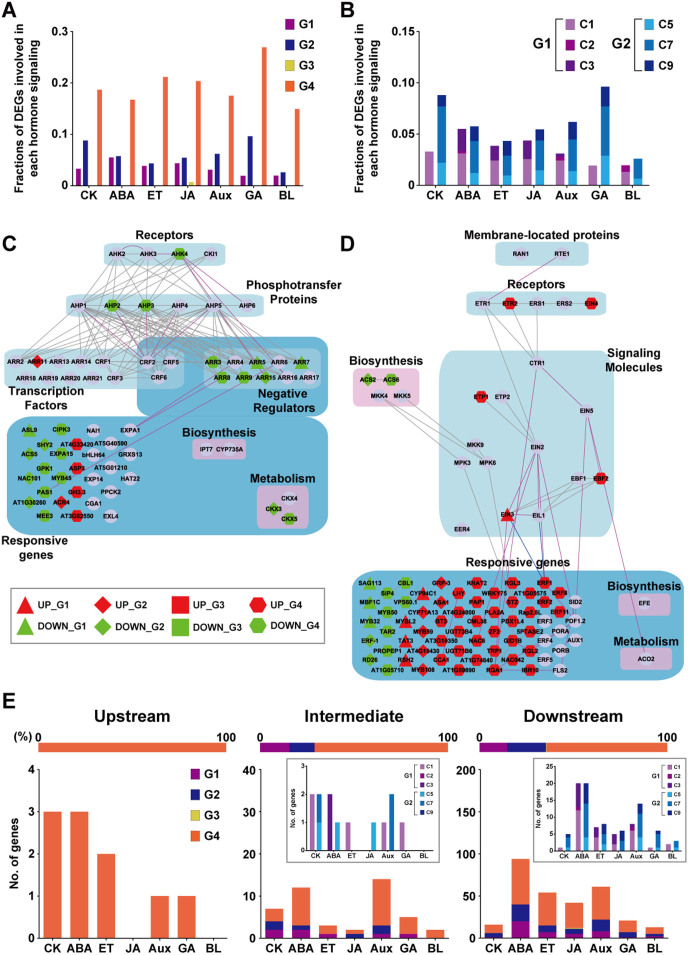
Extended regulatory structures contribute to robustness and diversification in regulation of hormone signaling networks. (A) Fractions of the genes in Groups 1–4 involved in seven hormone signaling networks. The fraction represents the number of the genes in each network divided by the total number of the genes in the network (Methods). CK, cytokinin; ABA, abscisic acid; ET, ethylene; JA, jasmonic acid; Aux, auxin; GA, gibberellin; and BL, brassinosteroid. (B) Distributions of the genes involved in the hormone signaling networks in Clusters 1–3 (C1–C3) in Group 1 and Clusters 5, 7, and 9 (C5, C7, and C9) in Group 2. (C–D) CK (C) and ET (D) signaling networks. Nodes were arranged into functional groups (blue and magenta backgrounds) based on their functions (e.g., receptors or transcription factors). Solid lines indicate PPIs (gray), genetic interactions (purple), or protein-DNA interactions (blue). Triangle, diamond, square, and hexagon nodes denote the genes in Groups 1–4, respectively (see node legend). Red and green nodes denote up- and down-regulated genes, respectively, while purple nodes denote the DEGs not included in Groups 1–4 or the genes in the signaling network with no expression changes in the network. (E) Numbers of signaling molecules in three layers (upstream molecules, intermediate signaling molecules, and downstream responsive genes) that belonged to Groups 1–4 in each hormone signaling network. For each layer of signaling molecules, the upper bar shows proportions of Groups 1–4 in the seven hormone signaling networks. The inlet stacked graph shows distributions of the genes involved in the hormone signaling networks in Clusters 1–3 (C1–C3) in Group 1 and Clusters 5, 7, and 9 (C5, C7, and C9) in Group 2.

Redundant genes were previously reported to be significantly enriched in signaling pathways, resulting in robustness and diversification in cellular signaling^4^. In plants, hormones control a broad spectrum of cellular processes associated with growth and development of plants^24^. Thus, we examined whether the extended regulatory structure by duplication of the ARR TFs contributed to robustness and diversification in hormone signaling. To this end, we counted how many molecules involved in signaling networks of seven representative hormones [cytokinin (CK), abscisic acid (ABA), ethylene (ET), jasmonic acid (JA), auxin (Aux), gibberellins (GA), and brassinosteroid (BL)] in *Arabidopsis* (Methods) belonged to Groups 1–4. For example, for ABA signaling, we obtained 418 genes involved in the ABA signaling network from previous literatures^25^,^26^ and GO database^27^. First, Groups 2 and 4 included 24 and 70 of the 418 genes, respectively (5.7 and 16.7% in Fig. 3A). Second, Clusters 1–3 in Group 1 included 13, 1, and 9 of the 418 genes, respectively (3.1, 0.2, and 2.2% in Fig. 3B). Third, Clusters 5, 7, and 9 in Group 2, which represent diversification of the genes redundantly regulated by two ARR TFs, included 5, 13, and 6 genes, respectively (1.2, 3.1, and 1.4% in Fig. 3B). Similarly, the ET, JA, Aux, and BL signaling networks included the molecules in Groups 2 and 4 (Fig. 3A), as well as Clusters 1–3 in Group 1 and Clusters 5, 7, and 9 in Group 2 (Fig. 3B). These data indicate that duplication of the ARR TFs contributes to robustness (Groups 2 and 4) and diversification (Clusters 1–3 of Group 1 and Clusters 5, 7, and 9 of Group 2) in the hormone signaling networks.

To further understand the effects of the extended regulatory structure on hormone signaling networks, we reconstructed network models for CK and ET signaling. The CK signaling network (Fig. 3C) was mainly represented by the down-regulated genes in the ARR deletion mutants (Fig. S3A). Interestingly, three upstream molecules in CK signaling (a CK receptor, WOL, and two phosphotransfer proteins, AHP2 and AHP3) belonged to Group 4 (1∨10∨12) (Fig. 3C). Moreover, three ARRs (ARR3/8/9), intermediate signaling molecules in CK signaling, belonged to Group 4. Furthermore, many downstream responsive genes (e.g., SHY2, ACS5, MEE3, and EXPA15) belonged to Group 2 or 4. All these data suggest that duplication of the ARR TFs can contribute to robustness in all the layers of CK signaling (upstream, intermediate, and downstream layers). On the other hand, the intermediate signaling layer included two ARRs (ARR5/7) belonging to Cluster 1 (Group 1), and two ARRs (ARR11/15) belonging to Clusters 5 and 7 (Group 2), respectively, unlike the upstream layer that included no DEGs belonging to Group 1 or 2. Moreover, the downstream layer included a responsive gene, ASL9, in Cluster 1, and four responsive genes (SHY2, ACS5, NAC101, and AT1G30260) in Clusters 5, 7, or 9 (Fig. S3B). These data suggest that duplication of the ARR TFs contributes to diversification mainly in the intermediate and downstream layers of hormone signaling.

Unlike the CK signaling network, the ET signaling network (Fig. 3D) was mainly represented by the up-regulated genes in the ARR deletion mutants (Fig. S3A). Nonetheless, similar to CK signaling, two upstream receptors (ETR2 and EIN4), two intermediate signaling molecules (ETP1 and EBF2), and four TFs (ERF1/2/8/11) in ET signaling belonged to Group 4 (Fig. 3D). Also, many downstream responsive genes belonged to Group 2 or 4. On the other hand, one intermediate signaling molecule (EIN3) belonged to Cluster 1, and four (CYP94C1, MYBL2, TAT3, and RSH2) and three (SAG113, MBF1C, and MYB32) downstream responsive genes belonged to Clusters 1 and 3, respectively (Fig. S3C). Also, eight responsive genes belonged to Clusters 5, 7, or 9. These data indicate that duplication of the ARR TFs contributes to robustness in all the layers of ethylene signaling, but diversification mainly in the intermediate and downstream layers. To examine whether these observations can be also seen in the other hormone signaling networks, we counted how many molecules in the three signaling layers (upstream, intermediate, and downstream) belonged to Groups 1–4, Clusters 1–3 in Group 1, and Clusters 5, 7, and 9 in Group 2 (Fig. 3E). The result showed that the molecules in all the signaling layers preferentially belonged to Group 2 or 4, while the molecules in the intermediate and downstream layers belonged to Clusters 1–3 or Clusters 5, 7, and 9 (Fig. 3E, inlet). All these data indicate that the extended regulatory structure by duplication of the ARR TFs contributed to robustness and diversification in hormone signaling.

### Extended regulatory structures contribute to robustness and diversification in functions of cellular networks of target genes

**Figure 4:**
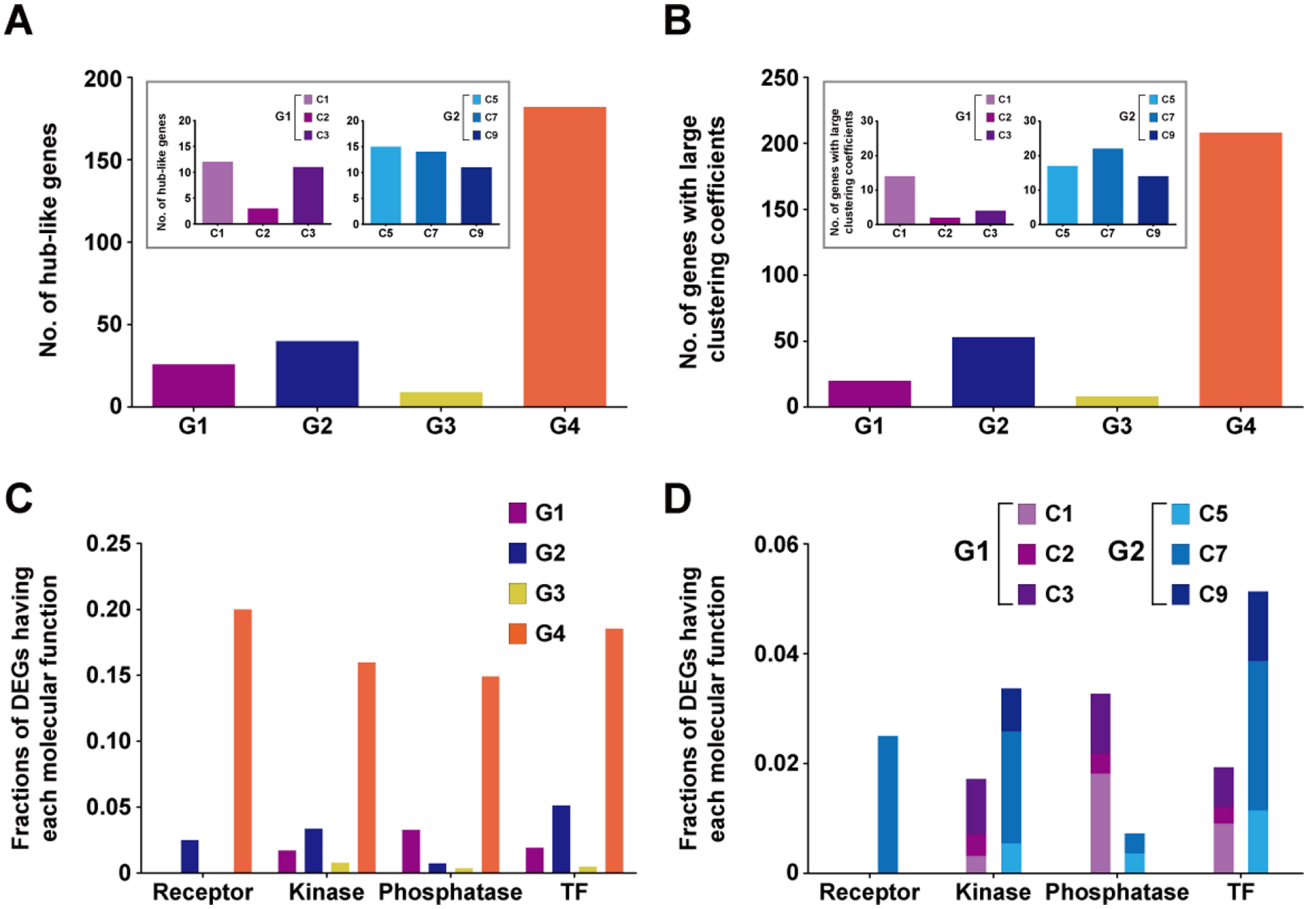
Extended regulatory structures contribute to robustness and diversification in regulation of cellular networks of target genes. (A–B) Numbers of hub-like DEGs (A) and DEGs with significantly large clustering coefficients (B) in Groups 1–4. The inlet stacked graph shows distributions of hub-like DEGs (A) and DEGs with large clustering coefficients (B) in Clusters 1–3 (C1–C3) in Group 1 and Clusters 5, 7, and 9 (C5, C7, and C9) in Group 2. (C) Fractions of the DEGs in Groups 1–4 with receptor, kinase, phosphatase, and TF activities. The fraction represents the number of the DEGs with the activity of each group of signaling molecules (e.g., receptor activity) divided by the total number of *Arabidopsis* genes with the same activity based on GOMF data. (D) Distributions of the DEGs with receptor, kinase, phosphatase, and TF activities in Clusters 1–3 (C1–C3) in Group 1 and Clusters 5, 7, and 9 (C5, C7, and C9) in Group 2.

The extended regulatory structures by duplication of the TFs can affect functions of cellular networks. To understand this notion, we next examined whether the extended regulatory structure affected functions of key molecules in cellular networks. First, a hub-like molecule with a large number of interactors critically affects functions of cellular networks^28^. Thus, we examined whether the extended regulatory structures by duplication of the ARR TFs affected regulation of hub-like molecules. To this end, among the DEGs in the ARR deletion mutants (Fig. 1A), we first identified 302 hub-like molecules (167 up-regulated and 135 down-regulated) using protein-protein interactome (PPI) data in iNID (Methods) and then counted how many the hub-like molecules belonged to Groups 1–4, Clusters 1–3 in Group 1, and Clusters 3, 5, and 7 in Group 2 (Fig. 4A). Many of the hub-like molecules belonged to Groups 2 and 4 with OR logics, suggesting that duplication of the ARR TFs contributes to robustness in regulation of the hub-like regulators. Furthermore, Clusters 1–3 included 12, 3, and 11 hub-like molecules, respectively, and Clusters 5, 7, and 9 included 15, 14, and 11 hub-like molecules, respectively (Fig. 4A, inlet), suggesting that duplication of the ARR TFs contributes to diversification in regulation of hub-like regulators.

Second, clustering coefficients for nodes represent how densely the 1^st^ neighbors of the nodes are connected. The nodes with large clustering coefficients can be highly influential in functions of the networks through dense connections with their neighbors. Thus, we examined whether the extended regulatory structures by duplication of the ARR TFs affected regulation of the nodes with significantly large clustering coefficients. To this end, among the DEGs in the ARR deletion mutants, we first identified 349 genes with large clustering coefficients (199 up-regulated and 150 down-regulated) (Methods) and then counted how many these genes belonged to Groups 1–4 (Fig. 4B). Many of these molecules belonged to Groups 2 and 4 with OR logics. Moreover, Clusters 1–3 included 14, 2, and 4 of these molecules, respectively, and Clusters 5, 7, and 9 included 17, 22, and 14 of these molecules, respectively (Fig. 4B, inlet). These data suggest that duplication of the ARR TFs can lead to robustness and diversification in regulation of these molecules with large clustering coefficients.

Third, we showed above that duplication of the TFs could lead to robustness and diversification in regulation of hormone signaling. Next, we further examined this feature in general cellular signaling networks including the hormone signaling networks. We first identified receptors, kinases, phosphatases, and TFs from the DEGs based on gene ontology molecular functions and counted how many of them belonged to Groups 1–4, Clusters 1–3 in Group 1, and Clusters 5, 7, and 9 in Group 2. These signaling molecules preferentially belonged to Group 4, followed by Group 2 (OR logics) (Fig. 4C). Moreover, kinases, phosphatases, and TFs belonged to Clusters 1–3 in Group 1, and also to Clusters 5, 7, and 9 (Fig. 4D). These data suggest that duplication of the ARR TFs contributes to robustness and diversification in regulation of cellular signaling networks. Taken together, all these data indicate that duplication of the ARR TFs contributes to robustness and diversification in regulation of key regulators, such as hub-like molecules, in cellular networks and thereby in functions of the cellular networks.

### Extended regulatory structures are utilized in responses to exogenous CK treatment

**Figure 5:**
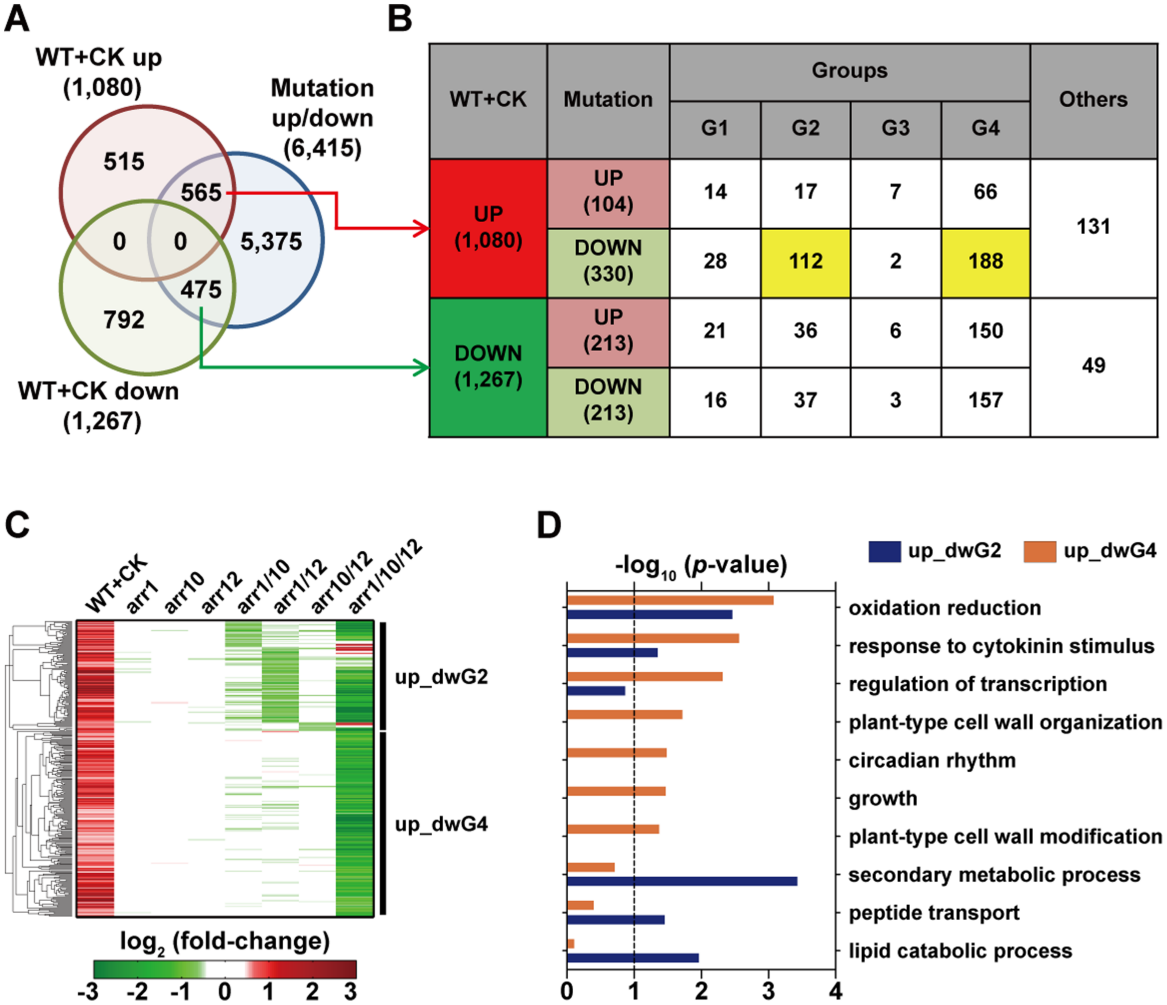
Extended regulatory structures are utilized in the responses to exogenous CK. (A–B) Venn diagram (A) and tabulation (B) showing relationships between the DEGs identified from the ARR deletion mutants (Mutation) and WT after CK treatment (WT + CK). UP, up-regulated genes; DOWN or dw, down-regulated genes. The numbers in parentheses are the numbers of the DEGs in each group. G1–4, Groups 1–4; Others, DEGs not belonging to Groups 1–4. (C) Genes up-regulated in WT by CK treatment and down-regulated genes in Groups 2 (up_dwG2) and 4 (up_dwG4) in the mutants. Green and red colors represent down- and up-regulation, respectively. Color bar shows the gradient of log_2_-fold-changes of gene expression levels in WT with and without CK treatment (1^st^ column) or gene expression levels between the ARR deletion mutants and WT (2^nd^–8^th^ columns). (D) GOBPs represented by up_dwG2 (blue) and up_dwG4 (orange). The bar represents −log_10_ (*p*-value) where *p*-value is the significance of the GOBPs being enriched by the genes in each group, which was computed from DAVID software.

The extended regulatory structures by duplication of the ARR TFs shown in Fig. 1B were identified under the natural condition where the endogenous level of CK was present in the system. Responses after the treatment of exogenous CK were often analyzed in plants to unveil the effects of CK on cellular processes^12^,^19^,^29^,^30^,^31^,^32^. We thus examined how the extended regulatory structures by the duplicated ARR TFs are utilized for the responses to external CK. To this end, we generated gene expression profiles of WT *Arabidopsis* roots treated with exogenous CK for 1 hour (Fig. S4A). By comparing gene expression levels between WT roots with and without CK treatment (WT + CK versus WT + mock), we identified 2,347 DEGs (1,080 up-regulated and 1,267 down-regulated genes) in CK-treated WT roots (Fig. 5A).

ARR1, 10, and 12 are positive regulators of CK signaling^19^,^20^,^21^,^31^. To examine how the CK responses are mediated by the duplicated *ARR* genes, we thus focused on the 1,080 up-regulated genes positively regulated by exogenous CK. Of the 1,080 up-regulated genes, 104 and 330 genes were up- and down-regulated, respectively, in the *ARR1, 10*, and/or *12* mutants (Fig. 5B). The up-regulated genes by exogenous CK treatment are expected to be down-regulated by deletion of the ARR TFs. Thus, of these two gene sets, we further focused on the 330 genes that showed the expected expression changes by exogenous CK treatment and in the ARR deletion mutants. Interestingly, these 330 genes belonged preferentially to Groups 2 (112 genes, up_dwG2 in Fig. 5C) and 4 (188 genes, up_dwG4 in Fig. 5C), both with OR logic regulatory structures, suggesting that the genes redundantly regulated by the ARR TFs were mainly utilized in the responses to exogenous CK. On the other hand, the ‘up_dwG2' genes included 26, 77, and 9 genes in Clusters 5, 7, and 9, respectively. Thus, this suggests that the redundantly regulated genes were further diversified when they were utilized in the responses to exogenous CK.

Furthermore, the ‘up_dwG2' genes were involved in secondary/lipid metabolic processes, and peptide transport. The ‘up_dwG4' genes were involved in growth, cell wall organization/modification, circadian rhythm, and regulation of transcription (Fig. 5D; Table S4). Also, the ‘up_dwG2' and ‘up_dwG4' genes were involved commonly in response to cytokinin stimulus and oxidation reduction. These data suggest that these cellular processes redundantly regulated by the ARR TFs are mainly utilized in the responses to exogenous CK. However, the ‘up_dwG2' genes involved in some of these processes included Clusters 5, 7, and 9 (Fig. S4B). For example, the ‘up_dwG2' genes involved in oxidation reduction 5, 7, and 1 genes in Clusters 5, 7, and 9, respectively. Thus, this suggests that diversification of the ‘up_dwG2' genes to Clusters 5, 7, and 9 is further utilized in regulation of the cellular processes in the responses to exogenous CK.

## Conclusions

Our knowledge of duplicated genes in signaling pathways has been limited in the roles of duplicated kinases in cellular signaling. A number of studies showed that duplicated kinases provide genetic buffering that leads to robustness in signaling systems against various perturbations and also diversification to acquire new functional or regulatory strategies over evolution^4^. Our approach provided new knowledge regarding the roles of duplicated TFs in the extension of transcriptional regulatory structures at the system level, leading to robustness and diversification in functions of target genes and in the regulation of the cellular networks of target genes. This knowledge can be used as a comprehensive basis to understand functions of TFs and cellular networks of target genes in signaling systems including duplicated TFs. Furthermore, our approach can be applied to other signaling systems in which TFs are duplicated and functional and regulatory roles of the TFs are still known.

## Methods

### Plant materials and growth conditions

*Arabidopsis thaliana* were grown in an environmentally controlled growth room (Korea Instruments, Seoul, Korea) at 22°C with continuous light. *Arabidopsis* seedlings were grown for 10 days on vertically oriented 1/2 MS agar plates. Root samples were obtained by cutting the plants at approximately the junction of root and hypocotyl with a sharp pincette. WT seedlings were either mock-treated or treated with 5 μM N^6^-benzyladenine (BA, Sigma B 3408) for 1 hour. The loss-of-function mutants of *arr1-3*, *arr10-5*, *arr12-1*, and *arr1-3/12-1* were isolated from the Salk T-DNA collection by using a PCR-based method. The *arr1-3*/*10-5*, *arr10-5*/*12-1*, and *arr1-3/10-5/12-1* were kindly provided by Dr. G. Eric Schaller^19^.

### RNA isolation and microarray experiment

Total RNA was isolated from root tissues using WelPrep™ (Welgene, Daegu, Korea) for microarray experiments. We then checked the integrity of the total RNA using a Bioanalyzer 2100 (Agilent, Santa Clara, CA, USA). The RNA integrity in all samples was sufficiently good for gene expression analysis (RNA integrity number >9.5). According to the standard Agilent protocols, the RNA was reverse-transcribed and amplified, and then hybridized onto the array (Agilent-031025 *Arabidopsis* 8 × 60 k), which includes 62,976 probes corresponding to 28,949 annotated genes (TAIR10). The mRNA levels were measured for two biological replicates for each plant: *arr1*, *arr10*, *arr12*, *arr1/10*, *arr10/12*, *arr1/12*, and *arr1/10/12* with no treatment; and WT (Col-0) with no treatment and with mock or CK treatment. The gene expression data set was deposited at the Gene Expression Omnibus database (GSE62597).

### Microarray analysis

Log_2_-intensities of the probes were first normalized using quantile normalization^33^. To identify DEGs, we then applied an integrative statistical method previously reported^22^ to the following comparisons: 1) untreated mutants (single, double, or triple mutant of *ARR1*, *10*, and *12* genes) versus untreated WT (Fig. 1A); and 2) CK-treated WT versus mock-treated WT (Fig. 5A). Briefly, for each gene, we calculated a T-statistic value using Student's *t*-test and also a log_2_-median-ratio in each comparison. We then estimated empirical distributions of T-statistics and log_2_-median-ratio for the null hypothesis (i.e. the genes are not differentially expressed) by random permutation experiments of all samples. Using the estimated empirical distributions, we computed adjusted *p*-values for the *t*-test and log_2_-median-ratio test for each gene and then combined these *p*-values with Stouffer's method^34^. Finally, we identified DEGs as the ones that have combined *p*-values ≤ 0.05 and absolute log_2_-median-ratios ≥ a cutoff value, 95^th^ percentile of the empirical distribution for log_2_-median-ratios in each comparison (e.g., log_2_-median-ratio = 0.51 for WT versus *arr1*).

### Selection and grouping of major regulatory structures

We first categorized the DEGs into total 239 Patterns based on their differential expression patterns in the single, double, and triple mutants, compared to WT (Fig. S5A). For each of the Patterns, we mapped a regulatory structure using AND and/or OR logics based on up- or down-regulation in single, double, and triple deletion mutations of *ARR1*, *10*, and *12* (Fig. S5B). The clusters mapped with the same regulatory structure were merged (Fig. S5B), and the clusters that could not be mapped to a regulatory structure due to the inconsistent differential expression patterns in the mutants were removed (Fig. S5C). This procedure resulted in the 15 regulatory structures for Clusters 1–15 in Fig. 1B. To select major regulatory structures, we computed 50^th^ percentiles of the sizes of the 15 clusters independently for up- and down-regulated genes (88 for up-regulated and 52 for down-regulated genes; Fig. S5D). We then selected the 10 clusters whose sizes were larger than 88 for up-regulated genes or 52 for down-regulated genes (Fig. 1B). Finally, we grouped them into 5 groups. For each group of the DEGs, the enrichment analysis of GOBPs was performed to identify cellular processes represented by the genes in the group using DAVID^23^. The GOBPs with *p* < 0.1 (a default cutoff) computed from DAVID were selected as the ones enriched by the genes in each group (Fig. 2B).

### Construction of hormone signaling networks

To construct hormone signaling networks, we first collected lists of hormone-related genes from Gene Ontology Biological Process (GOBP) data^27^. Hormone-related genes are defined as the ones annotated with at least one of the following terms: for example, in the case of cytokinin, ‘response to cytokinin', ‘cytokinin biosynthetic process', ‘cytokinin metabolic process', ‘cytokinin transport', and ‘cytokinin-activated signaling pathway'. We then constructed the seven hormone signaling networks with those hormone-related genes based on KEGG pathway^35^, Science Signaling^36^,^37^,^38^, iNID^39^, and previous literatures^25^,^26^,^40^,^41^,^42^.

### Identification of hub-like molecules and the nodes with large clustering coefficients

To identify hub-like molecules, we first calculated the number of interactors (degree) for each protein using PPIs in iNID^39^. We then estimated the empirical distribution of the degree by randomly sampling 100,000 proteins from the whole annotated proteins. Using the estimated empirical distribution, we computed *p*-values for each protein and the proteins with *p* < 0.05 were defined as hub-like molecules. To identify nodes with significantly large clustering coefficients, we performed the same analysis described above, but the empirical distribution and *p*-values was estimated for clustering coefficient, and the genes with *p* < 0.05 were selected as the nodes with significantly large clustering coefficients. Clustering coefficients for the DEGs were computed using Network Analyzer (Release 2.7)^43^ in Cytoscape.

### Analysis of signaling networks

To analyze signaling networks, we categorized the signaling molecules in the DEGs into receptors, kinases, phosphatases, and transcription factors based on gene ontology molecular function (GOMF)^27^ and then obtained the DEGs with the activity of each group of the signaling molecules as the ones with the corresponding GOMF term (e.g., receptor activity).

## Author Contributions

D.H. and H.G.N. designed the experiments and analyses; S.H.C., I.H.L., S.J.P. and I.C.L. performed the experiments; D.Y.H., S.H. and S.H.C. performed data analyses; and S.H.C., D.Y.H., D.H. and H.G.N. wrote the manuscript. All authors reviewed the manuscript.

## Supplementary Material

Supplementary Information Supplementary Information

Supplementary Information Dataset 1

Supplementary Information Dataset 2

Supplementary Information Dataset 3

Supplementary Information Dataset 4
