# Supplementary material for: Gene duplication of type-B ARR transcription factors systematically extends transcriptional regulatory structures in Arabidopsis
Source: Sci Rep. 2014 Nov 26;4:7197. doi: 10.1038/srep07197 (PMC4244629; doi:10.1038/srep07197)
Supplement: Supplementary Information [file srep07197-s1.pdf]

Supplementary Information for

Gene duplication of type-B ARR transcription factors systematically extends transcriptional regulatory structures in *Arabidopsis*

Seung Hee Choi[1,*], Do Young Hyeon[2,*], Il Hwan Lee[1], Su Jin Park[2], Seungmin Han[2], In Chul Lee[3], Daehee Hwang[2,3,4] & Hong Gil Nam[3,4]

[1]Division of Molecular and Life Sciences, POSTECH, Pohang 790-784, Republic of Korea; [2]School of Interdisciplinary Bioscience and Bioengineering, POSTECH, Pohang 790-784, Republic of Korea; [3]Center for Plant Aging Research, Institute for Basic Science, DGIST, Daegu 711-873, Republic of Korea; and [4]Department of New Biology, DGIST, Daegu 711-873, Republic of Korea.

*These authors equally contributed to this work.

Correspondence and requests for materials should be addressed to D.H. (dhwang@dgist.ac.kr) or H.N. (nam@dgist.ac.kr)

This PDF file includes:

Figs. S1 to S5

Other Supplementary information for this manuscript includes the following:

Tables S1-4 (as Excel files)

Figures

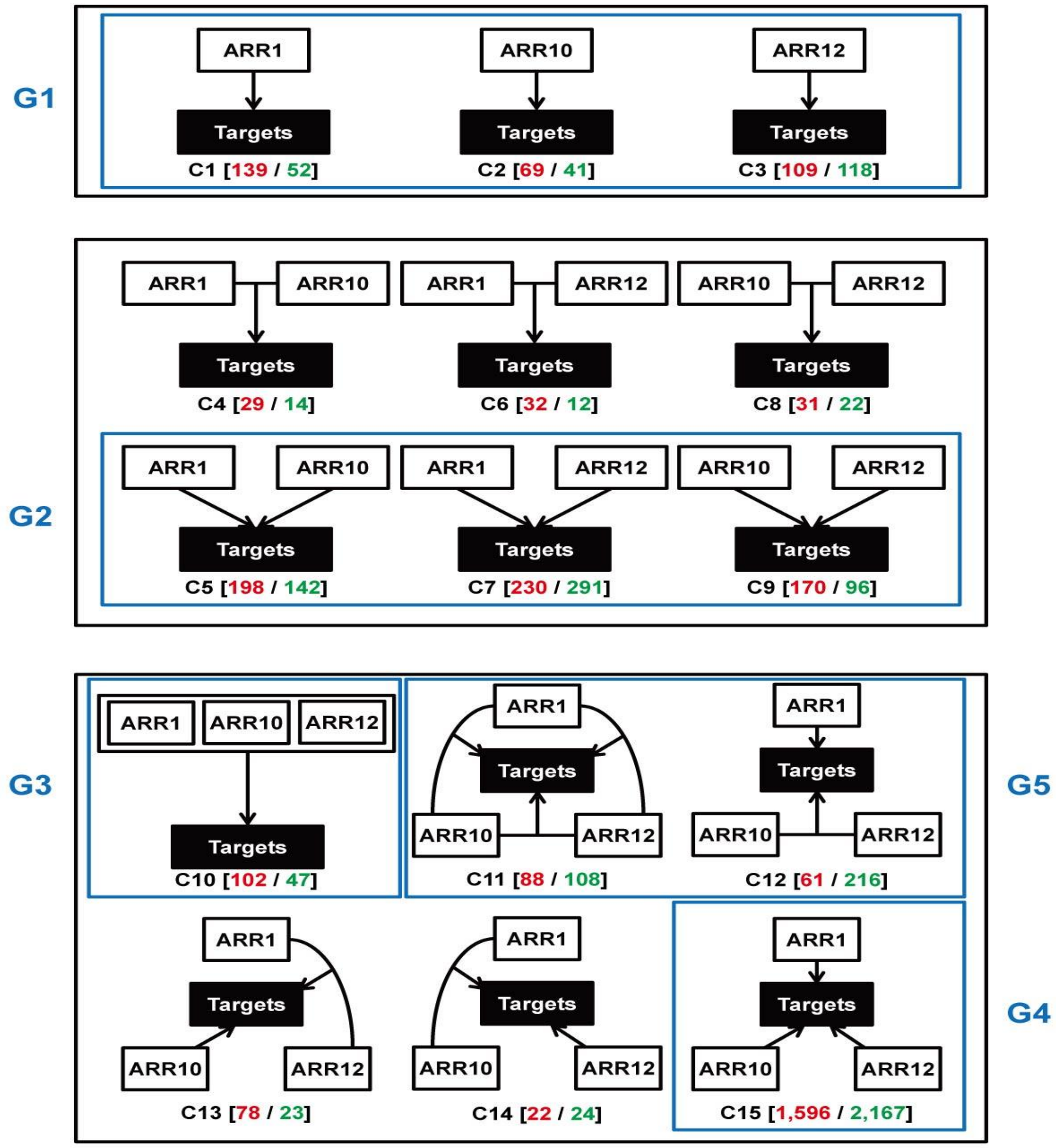

Fig. S1. 15 transcriptional regulatory structures extended by duplication of *ARR1*, *10*, and *12*. The regulatory structures correspond to the 15 clusters of the DEGs shown in **Fig. 1B** (see C1 to C15 below the schematic diagrams describing the regulatory structures). AND ('∧') and OR gates ('∨') were denoted by the joined and independent arrows, respectively. The five major regulatory structures (G1-5 in **Fig. 1B**) were indicated by the blue boxes. The numbers in the brackets, next to C1-15, represent the numbers of the up- (red) and down- (green) regulated genes belonging in each cluster.

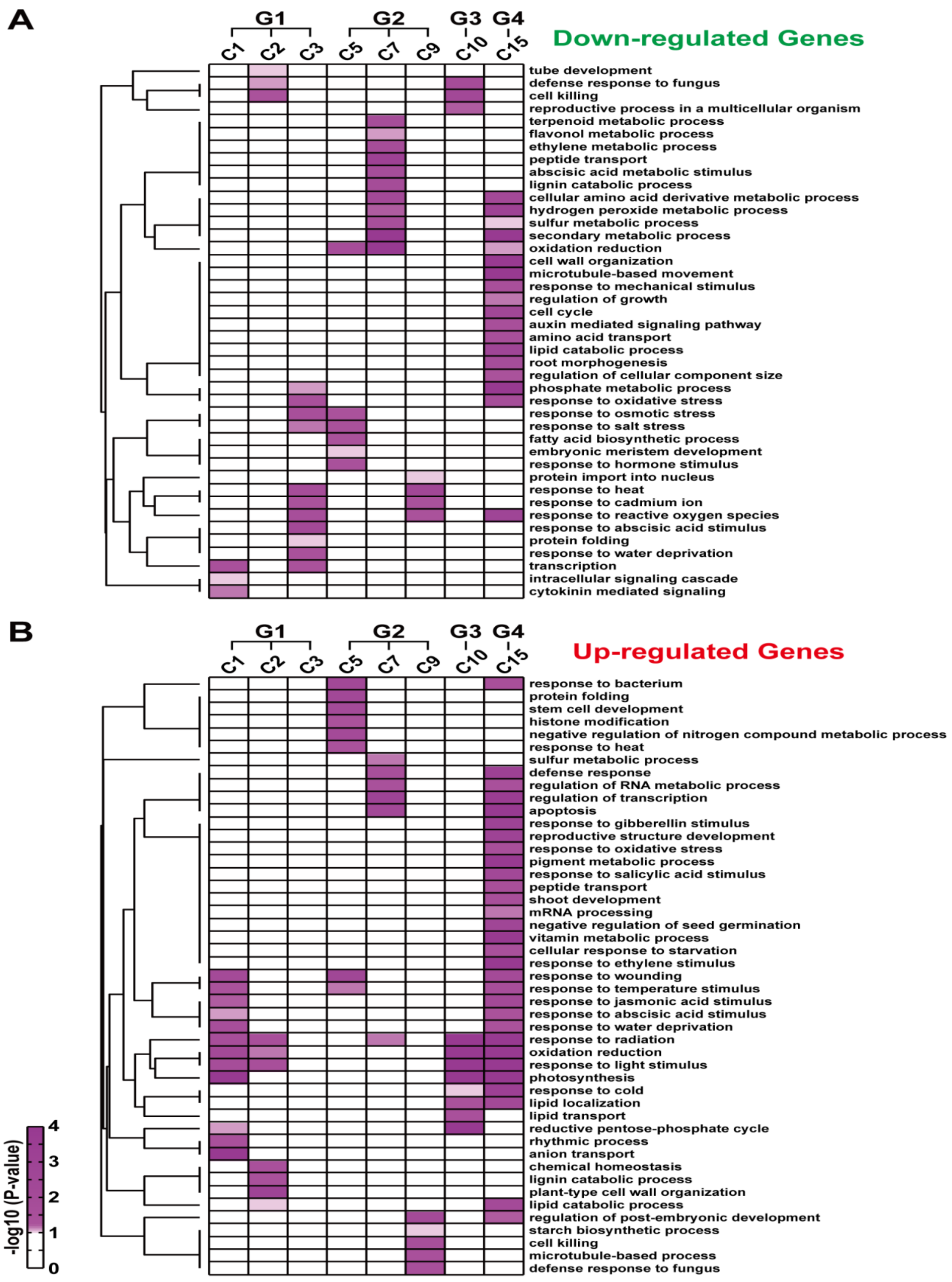

Fig. S2. GOBPs represented by the down- (upper) and up-regulated genes (lower) in the clusters of Groups 1-4 (Fig. 1B). The color bar represents the gradient of -$\log_{10}$ (*p*-value) where *p*-value is the significance of the GOBPs being enriched by the genes in each cluster, which was computed from DAVID software. The GOBPs in the heat map were clustered using the -$\log_{10}$ (*p*-value) by a hierarchical clustering method (average linkage and Euclidean distance as similarity measure).

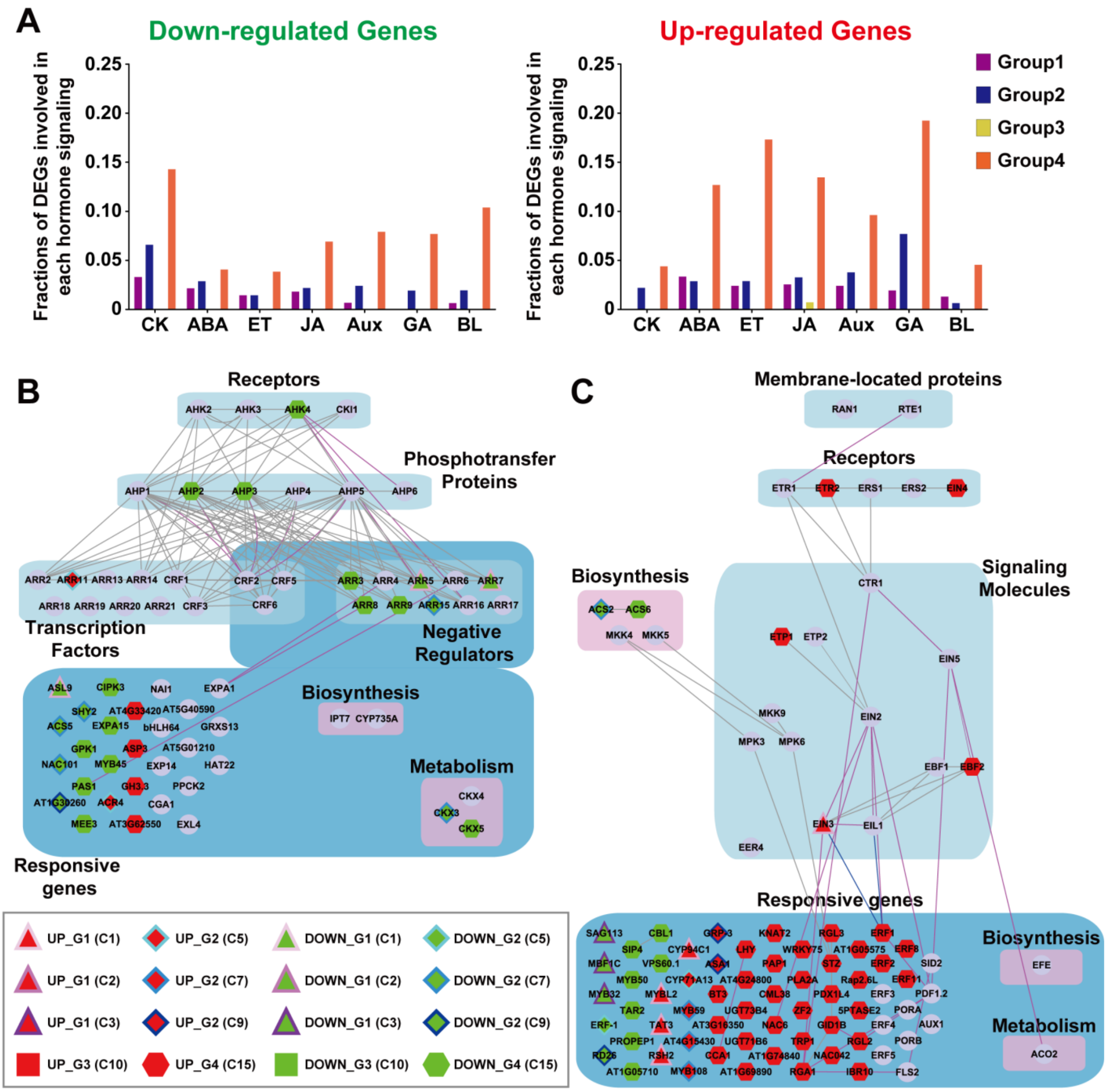

Fig. S3. Extended regulatory structures contribute to robustness and diversification in regulation of hormone signaling networks. (A) Fractions of down- (left) and up-regulated genes (right) in Groups 1-4 involved in seven hormone signaling networks. The fraction represents the number of up- or down-regulated genes in each network divided by the total number of the genes in the network (Methods). CK, cytokinin; ABA, abscisic acid; ET, ethylene; JA, jasmonic acid; Aux, auxin; GA, gibberellin; and BL, brassinosteroid. (B-C) Cytokinin (B) and ethylene (C) signaling networks. Nodes were arranged into functional groups (blue and magenta backgrounds) based on their functions (e.g., receptors or transcription factors). Triangle, diamond, square, and hexagon nodes denote the genes in Groups 1-4, respectively (see node legend). Node boundary colors denote the genes in Clusters 1-3 in Group 1 (purple) and Clusters 5, 7, and 9 in Group 2 (blue; see node legend). Red and green nodes denote up- and down-regulated genes, respectively, while purple nodes denote the DEGs not included in Groups 1-4 or the genes in the signaling network with no expression changes in the network. Solid lines indicate PPIs (gray), genetic interactions (purple), or protein-DNA interactions (blue).

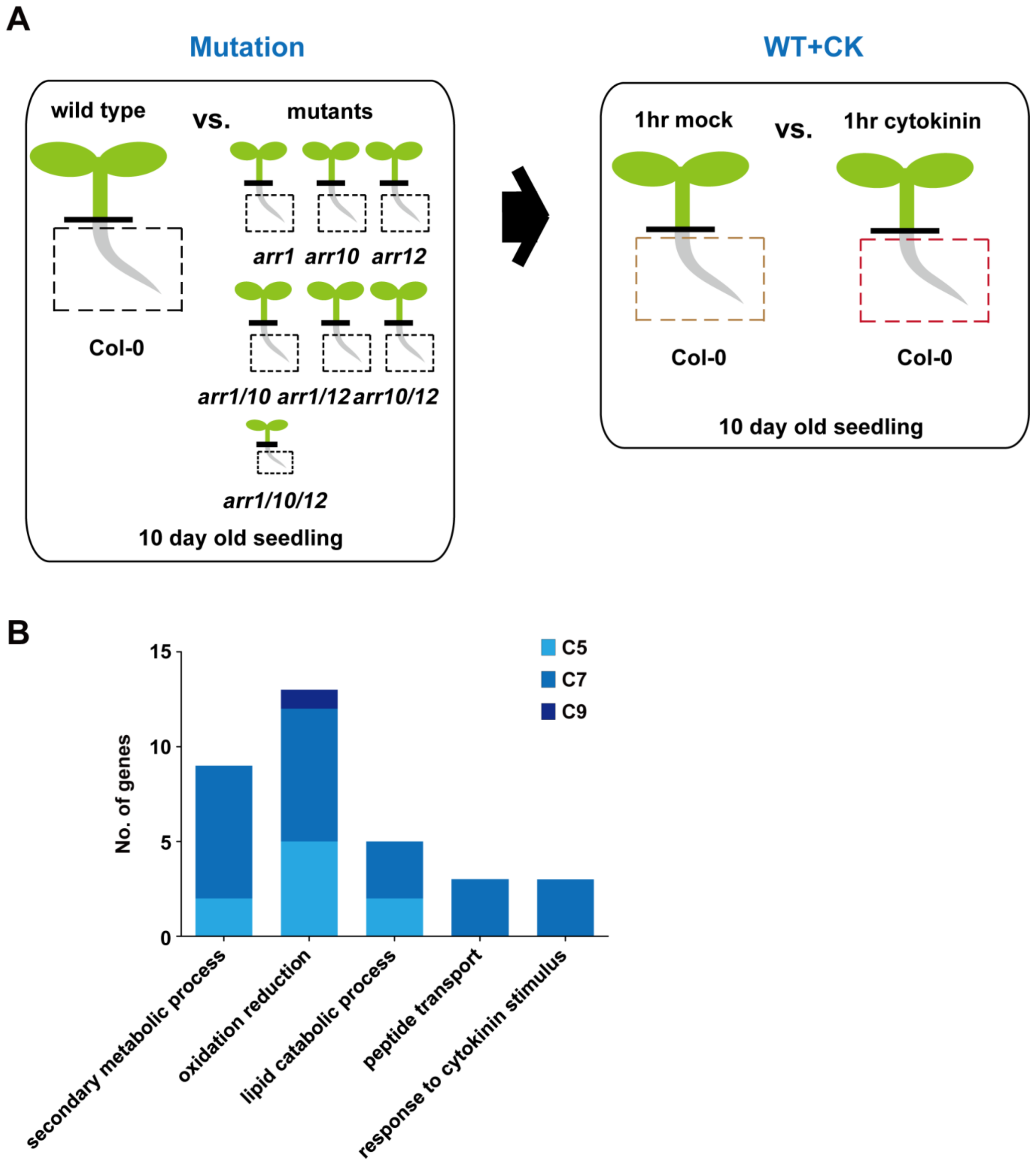

Fig. S4. Extended regulatory structures are utilized in the responses to exogenous cytokinin. (A) Schematics showing the experimental procedure for mock and cytokinin treatment. Gene expression profiles were generated from the roots of 10 day old seedling of the following two groups of plants: 1) WT plants and single (*arr1*, *arr10*, and *arr12*), double (*arr1/10*, *arr1/12*, and *arr10/12*), and triple deletion mutants (*arr1/10/12*) with no treatment (left box); 2) WT plants at 1 hour after mock and cytokinin treatments (right box). (B) Distributions of the genes involved in the GOBPs represented by the 'up_dwG2' genes (Fig. 5C) in Clusters 5, 7, and 9 in Group 2.

A

Pattern	arr1	arr10	arr12	arr1/10	arr1/12	arr10/12	arr1/10/12	Count
1	N	N	N	N	N	N	U	1,596
2	N	N	N	U	N	N	N	173
3	N	N	N	N	U	N	N	152
4	N	N	N	N	N	U	N	149
5	N	N	U	N	N	N	N	78
6	N	N	N	N	U	N	U	70
7	N	N	N	U	N	U	N	65
8	N	N	N	U	U	U	N	58
9	U	N	N	N	N	N	N	51
10	N	U	N	N	N	N	N	50
11	N	N	N	U	U	N	N	47
12	U	N	N	N	N	N	U	36
13	N	N	N	U	U	U	U	29
14	U	U	U	U	U	U	N	21
15	U	N	N	N	U	N	U	20
16	U	U	U	N	N	N	N	19
17	N	N	N	U	N	N	U	16
18	N	N	N	N	N	U	U	16
19	N	N	N	N	U	U	N	16
20	N	N	N	U	U	N	U	13
21	N	U	U	N	N	N	N	11
22	U	U	N	N	N	N	N	10
23	U	U	U	N	N	N	U	10
24	U	U	U	N	N	N	D	10
25	N	N	N	U	N	U	U	10
26	U	N	N	U	U	N	N	9
27	N	U	N	N	N	N	D	9
28	N	N	N	U	N	N	D	9
29	N	U	N	N	N	N	U	8
30	N	N	U	N	N	U	N	8

•
•
•

B

Pattern	arr1	arr10	arr12	arr1/10	arr1/12	arr10/12	arr1/10/12	Count	Regulatory Logic	Cluster
2	N	N	N	U	N	N	N	173	1∨10	C5
17	N	N	N	U	N	N	U	16	1∨10	
28	N	N	N	U	N	N	D	9	1∨10	
3	N	N	N	N	U	N	N	152	1∨12	C7
6	N	N	N	N	U	N	U	70	1∨12	
31	N	N	N	N	U	N	D	8	1∨12	
4	N	N	N	N	N	U	N	149	10∨12	C9
18	N	N	N	N	N	U	U	16	10∨12	
43	N	N	N	N	N	U	D	5	10∨12	

C

Pattern	arr1	arr10	arr12	arr1/10	arr1/12	arr10/12	arr1/10/12	Count
186	N	N	U	U	U	U	N	13
187	U	N	N	U	U	U	U	7
188	N	N	U	U	N	N	N	5
189	U	N	N	N	U	U	U	3
190	N	N	U	U	N	U	N	3
191	N	N	U	U	N	U	U	2
192	U	N	N	N	N	U	N	1
193	U	N	N	N	U	U	N	1
194	U	N	N	N	N	U	U	1
195	U	N	N	U	N	U	U	1

D

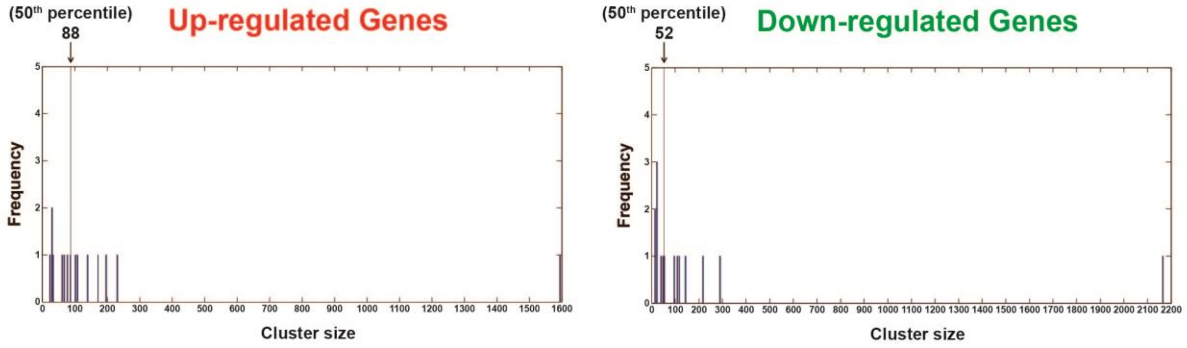

Fig. S5. **Selection and grouping of major regulatory structures.** (A) Patterns of the DEGs based on their differential expression patterns in the single (*arr1*, *10*, and *12*), double (*arr1/10*, *arr1/12*, and *arr10/12*), and triple (*arr1/10/12*) mutants of *ARR1*, *10*, and *12*, compared to WT. Green and red colors represent down- and up-regulation, respectively. U, up-regulation; D, down-regulation; and N, no change. Counts denote the numbers of the DEGs that belonged to the corresponding Patterns. Only the largest 30 Patterns were presented. (B) Mapping a regulatory structure using AND and/or OR logics ('Regulatory Logic' column) to each Pattern and merging the Patterns with the same regulatory structure into a cluster of the DEGs ('Cluster'). Nine example Patterns merged into C5-9 were shown. (C) Removed clusters that could not be mapped to a regulatory structure due to the inconsistent differential expression patterns in the mutants. Ten example Patterns removed were shown. (D) Distributions of the cluster sizes for up- (left) and down-regulated genes (right). The 50th percentiles (88 and 52 for up- and down-regulated genes) used as the cutoff values in selection of major regulatory structures were denoted.
